# Sevoflurane Inhibits Proliferation, Invasion, but Enhances Apoptosis of Lung Cancer Cells by Wnt/β-catenin Signaling via Regulating lncRNA PCAT6/miR-326 Axis

**DOI:** 10.1515/biol-2020-0017

**Published:** 2020-04-10

**Authors:** Guoning Su, Zhibing Yan, Min Deng

**Affiliations:** 1 Department of Anesthesiology, Yunnan Second People's Hospital, No.176 Qingnian Road, Kunming, Yunan, 652600, China; 2 Department of Anesthesiology, Jiangmen Central Hospital, Jiangmen, Guangdong 529030, China

**Keywords:** lung cancer, sevoflurane, lncRNA PCAT6, miR-326, Wnt5a/β-catenin signaling

## Abstract

Sevoflurane was frequently used as a volatile anesthetic in cancer surgery. However, the potential mechanism of sevoflurane on lung cancer remains largely unclear. In this study, lung cancer cell lines (H446 and H1975) were treated by various concentrations of sevoflurane. 3-(4,5-dimethyl-2-thiazolyl)-2,5-diphenyl-2-H-tetrazolium bromide (MTT) assessment and colony formation assay were performed to detect the cell viability and proliferation, separately. Also, transwell assay or flow cytometry assay was applied as well to evaluate the invasive ability or apoptosis in lung cancer cells, respectively. Western blot assay was employed to detect the protein levels of β-catenin and Wnt5a. Moreover, quantitative real-time polymerase chain reaction (qRT-PCR) was used to examine the expression level of prostate cancer-associated transcript 6 (PCAT6) and miR-326 in lung cancer tissues and cells. The target interaction between miR-326 and PCAT6 or Wnt5a was predicted by bioinformatics analysis and verified by the dual-luciferase reporter gene assay. Sevoflurane inhibited the abilities on viability, proliferation, invasion, and activation of Wnt/β-catenin signaling, but promoted apoptosis of H446 and H1975 cells in a dose-dependent manner. The expression of PCAT6 was increased in lung cancer tissues and cells, except for that of miR-326. Besides, sevoflurane could lead to expressed limitation of PCAT6 or improvement of miR-326. This process presented a stepwise manner. Up-regulation of PCAT6 restored the suppression of sevoflurane on abilities of proliferation, invasion, rather than apoptosis, and re-activated the Wnt5a/β-catenin signaling in cells. Moreover, the putative binding sites between miR-326 and PCTA6 or Wnt5a were predicted by starBase v2.0 software online. PCAT6 suppressing effects on cells could be reversed by pre-treatment with miR-326 vector. The promotion of Wnt5a inverted effects led from miR-326 or sevoflurane. Our study indicated that sevoflurane inhibited the proliferation, and invasion, but enhanced the apoptosis in lung cancer cells by regulating the lncRNA PCAT6/miR-326/Wnt5a/β-catenin axis.

## 1 Introduction

Sevoflurane was one of the frequently-used anesthetics in tumor resection. Related studies have shown that Sevoflurane’s inhibitory effect on cancer cells has been confirmed in a variety of tumors including lung cancer [1, 2, 3]. Besides, it has been shown to inhibit cell growth, invasion, and migration, triggering morphological changes and apoptosis by regulating changes of long noncoding RNAs (lncRNAs) in several types of cancer cell lines, including lung cancer cells [1, 4, 5, 6]. LncRNAs were non-coding RNA and acted as regulators to participate in proliferation, migration, invasion, apoptosis and stem cell pluripotency by affecting target gene expression in transcriptionally or post-transcriptionally [7, 8]. Currently, dysregulated expression of lncRNAs was observed in most cancers, which was closely associated with a poor prognosis of cancer patients [9, 10, 11]. It has been reported that lncRNAs were platforms and essential regulators between RNAs and cancers [12].

LncRNA prostate cancer-associated transcript 6 (PCAT6), located at chromosome 1q32.1, has been proven to function as oncogenic lncRNA in tumors [13, 14]. Studies have shown that lncRNA PCAT6 is up-regulated in lung cancer tissues and cells and is associated with tumor progression [15, 16]. Wan *et al*. explored the potential regulatory mechanisms of PCAT6 on proliferation, colony formation, and apoptosis in lung cancer and indicated that PCAT6 was closely associated with a poor prognosis [15]. Li *et al*. reported that knockdown of PCAT6 might be a potential therapeutic strategy for lung adenocarcinoma [17], but further studies on a more detailed understanding of the underlying molecular mechanisms of PCAT6 were needed. We explored expression of different cancer-related lncRNAs in lung cancer cells after sevoflurane inducement, namely PCAT6, Urothelial cancer associated 1 (UCA1), Small Nucleolar RNA Host Gene 1 (SNHG1), colon cancer-associated transcript 2 (CCAT2), and found that PCAT6 was the only one significantly suppressed after sevoflurane administration. Whether sevoflurane can regulate PCAT6 expression in lung cancer, and thus affect the malignant biological behavior of tumor cells has not been reported.

In addition, accumulating evidence has indicated miRNAs acted as regulators by regulating oncogene expression in malignant cancers [18, 19, 20]. The latest researches showed that miR-326 was down-regulated in lung cancer tissues and cells, and the pattern of miR-326, exerted a tumor suppressor, could reverse cell proliferation, invasion, cell cycle, and resistance in lung cancer cells [21, 22, 23]. A previous finding revealed that miRNA-326 inhibited proliferation and invasion of non-small cell lung cancer via targeting Notch homolog 1 [24]. Another study investigated by Wang *et al*., discovered downregulation of miR-326 in lung cancer cells, further research indicated that upregulation of miR-326 suppressed proliferation, invasion, migration and induced cell apoptosis, cycle arrest of lung cancer *in vitro* by downregulating Specificity protein 1 transcription factors and inactivation of JAK2/STAT3 and PI3K/AKT signaling pathways [21]. In our research, through the prediction of bioinformatics, PCAT6 has a significant correlation with miR-326 among multiple miRNAs (miR-326, miR-545-3p, miR-185-3p, miR-330-5p, miR-143, miR-513a-5p) targeted by PCAT6. Nevertheless, the regulatory role of miR-326 in lung cancer has not been fully clarified.

The Wnt/β-catenin signaling pathway has been implicated in a wide range of physiological and pathophysiological cancer processes, including lung cancer [25, 26, 27, 28, 29]. Tan *et al*. found that the expression of miR-625 inhibited the proliferation, apoptosis, migration, and invasion *in vitro* by directly targeting homeobox B5 and deactivating the Wnt/β-catenin pathway [30]. Gao *et al*. confirmed that miRNA-378 promoted the progression of lung cancer by inactivation of Wnt/β-catenin and ERK1/2 pathways [31].

Given the above studies, this study was designed to better understand the molecular mechanism of sevoflurane inhibiting lung cancer progression, and determining relationships among PCAT6, miR-326, and the Wnt/β-catenin pathway.

## 2 Materials and methods

### 2.1 Samples collection

45 pairs of lung cancer tissues and adjacent normal lung tissues were obtained from patients who underwent surgery at Yunnan Second People’s Hospital. Clinicopathological characteristics of patients are presented in Table 1. All tissue samples were stored at -80°C conditions.

**Table 1:** Clinicopathological characteristics of patients with lung cancer

Characteristic	N
Gender	
male	26
female	19
Age（years）	
≥60	31
＜60	14
Tumor stage	
Ⅰ-Ⅱ	20
Ⅲ	25
Metastasis	
No	24
Yes	21

The inclusion criteria were: Patients with complete clinical data; Patients had not received chemotherapy, radiotherapy, or immuno-therapy before surgery. The exclusion criteria were: Lack of clinical data; Area of necrosis in the tumor tissue > 60%; and Presence of additional malignant tumors.

**Informed consent**: Informed consent has been obtained from all individuals included in this study.

**Ethical approval**: The research related to human use has been complied with all the relevant national regulations, institutional policies and in accordance the tenets of the Helsinki Declaration, and has been approved by the Ethics Committee of Yunnan Second People’s Hospital.

### 2.2 Cell lines, cell culture, and sevoflurane administration

Human bronchial epithelial cell lines (16HBE) and two different lung cancer cell lines (H446 and H1975) were purchased from Shanghai Innovation Biotechnology Co., Ltd. (Shanghai, China). All cells were cultured in 90% Dulbecco’s modified Eagle’s medium (DMEM; Gibco, Grand Island, NY, USA) with 10% fetal Bovine Serum (FBS; Gibco) at 37°C condition with 5% CO_2_. The H446 and H1975 cells were divided into 4 groups: control group (without sevoflurane), 1.7% sevoflurane group, 3.4% sevoflurane group, and 5.1% sevoflurane group. H446 and H1975 cells were exposed to 3.4% sevoflurane for 6 hours for subsequent experiments. Cells in log phase were seeded onto plates and incubated for 24 hours at 37°C. The cell culture plate was then placed at the inlet and connected to a sealed glass chamber of an anesthesia device (Cicero-EM 8060; Drager, Lübeck, Germany), and the other chamber was a gas monitor (PM8060; Drager) to monitor the concentration of sevoflurane. The anesthesia machine was connected to an anesthetic vaporizer (Sevorane; Abbott, Abbot Park, IL, USA) to supply sevoflurane. After treatment with sevoflurane, the cells were incubated for an additional 24 hours at 37°C and then used for subsequent functional and molecular biology experiments.

### 2.3 3-(4,5-dimethyl-2-thiazolyl)-2,5-diphenyl-2-H-tetrazolium bromide (MTT)

The cell viability of lung cancer cells was evaluated through MTT assay. Briefly, H446 and H1975 cells (3×103/ well) with or without transfection were seeded into 96-well plates (Corning Inc., Corning, NY, USA) and maintained in an incubator with 5% CO_2_ at 37˚C for 24 h. Then, MTT (20 μL) from Sigma (St Louis, MO, USA) was added to each well and kept for 4 h. Following this, the supernatant of each well was discarded, and DMSO (150 μL, Sigma) was added for the dissolution of the formazan crystals. In the end, the Microplate Absorbance Reader (Thermo Fisher Scientific, Waltham, MA, USA) was utilized for the assessment of the optical density value at 490 nm.

### 2.4 Colony formation assay

H446 and H1975 cells were placed in 6-well plates overnight and then transfected with indicated vectors, and then exposed to the dose of 3.4% sevoflurane. After stimulation for 6 hours, the medium was changed with fresh medium and updated every three days. Two weeks later, H446 and H1975 cells were carefully washed with pre-cold phosphate-buffered saline (PBS) and fixed using the paraformaldehyde (4%, 1 mL) for 30 min at 4°C. Subsequently, the cells were washed by PBS and stained with 0.1% crystal violet for 1 hour, and again washed with PBS until per well was clear. If a colony exceeded 50 cells, the colony was counted.

### 2.5 Transwell assay

The rate of cell invasion was investigated by the transwell chamber (Corning Inc.) with matrigel matrix. The lower chamber was added with DMEM medium with 10% FBS, while the transfected H446 and H1975 cells were injected into the upper one with 100 μL of serum-free medium, and the whole steps were carried out according to the manufacturer’s instructions. In the end, paraformaldehyde (PFA; Sigma) was used to attach cells on the lower surface of the upper chamber. Cells were analyzed under a microscope after staining with crystal violet.

### 2.6 Flow cytometry assay

Annexin V-FITC/PI Apoptosis Detection Kit (Vazyme, Nanjing, China) was used for flow cytometry assay. In brief, H446 and H1975 cells with different transfection were seeded into 6-well plates, treated with 0.25% trypsin and washed with pre-cooled PBS. Thereafter, cells (2×105) were resuspended by 100 μL 1 × binding buffer and then incubated by 5 μL Annexin V-fluorescein isothiocyanate (FITC) and propidium iodide (PI) staining solution for 10 min at room temperature without light. Finally, the apoptotic cells were analyzed using flow cytometry (BD Biosciences, San Jose, CA, USA).

### 2.7 Western blot

The protocol was operated in accordance with the descriptions in a previous paper [32]. Briefly, the segregated proteins were blotted on the membranes (Polyvinylidene Fluoride; Millipore, Bedford, MA, USA). Then, the membranes were incubated with unique primary antibodies overnight at 4°C after blocking for 1 hour. On the next day, the corresponding secondary antibody was used for the combination with the primary antibody, and the combined signals were appeared via adding the reagents of an enhanced chemiluminescence kit (Millipore). The primary antibodies against c-Myc (ab168727, 1:1000, Abcam, Cambridge, UK), Matrix metallopeptidase 9, (MMP-9, ab137867, 1:1000, Abcam), Ceaved-caspase-3 (ab49822, 1:1000, Abcam), Wnt5a (ab174963, 1:1000, Abcam), β-catenin (ab16051, 1:1000, Abcam), β-actin (ab179467, 1:2500, Abcam), and goat anti-rabbit secondary antibodies (ab205718, 1:5000, Abcam) were used in this study.

### 2.8 Quantitative real-time polymerase chain reaction (qRT-PCR)

Total RNAs from H446 and H1975 cells were extracted through TRIzol reagent (Life Technologies Corporation, Carlsbad, CA, USA). Primer-Script one-step RT-PCR kit (Takara, Shiga, Japan) or miRNA Reverse Transcription kit (GeneCopoeia, FulenGen, China) was employed to synthesize the first-strand complementary DNA of PCAT6 or miR-326, individually. The levels of PCAT6 and miR-326 were assessed via SYBR Premix Dimer Eraser Kit (Takara). The primers sequences used were presented as below:

PCAT6 forward (5’-CAGGAACCCCCTCCTTACTC-3’),

PCAT6 reverse (5’-CTAGGGATGTGTCCGAAGGA-3’),

miR-326 forward (5’-ACTGTCCTTCCCTCTGGGC-3’),

miR-326 reverse (5’-AATGGTTGTTCTCCACTCTCTCTC-3’),

U6 forward (5’-GCTTCGGCAGCACATATACTAAAAT-3’),

U6 reverse (5’-CGCTTCACGAATTTGCGTGTCAT-3’),

β-actin forward (5’-ATCACCATTGGCAATGAGCG-3’),

β-actin reverse (5’-TTGAAGGTAGTTTCGTGGAT-3’).

The expression levels of PCAT6 and miR-326 were calculated by the 2-^ΔΔCt^ method, and β-actin or U6 snRNA was viewed as an internal control for PCAT6 or miR-326, respectively.

### 2.9 Cell transfection

For lncRNA downregulation, small interfering RNA (siRNA) against PCAT6 (si-PCAT6) and its negative control (si-NC) were constructed by Genepharma (Shanghai, China). For PCAT6 upregulation, PCAT6 sequences were amplified by PCR and inserted into pcDNA vector (Invitrogen, Carlsbad, CA, USA) to generate fusion plasmids, named as PCAT6, and pcDNA empty vector (vector) as the control. For miR-326 enrichment or inhibition, miR-326 mimics (miR-326), miR-326 inhibitor (anti-miR-326), and its own negative control (miR-NC and anti-NC) were purchased from Ribobio (Guangzhou, China). For Wnt5a overexpression, Wnt5a sequences were amplified by PCR and inserted into pcDNA vector (Invitrogen) to generate fusion plasmids, namely Wnt5a, pcDNA empty vector (vector) as the control. For each well, equal doses (100 pmol) of miRNA mimics, inhibitors, or negative control RNAs were used. All items were introduced into H446 and H1975 cells using Lipofectamine 3000 (Invitrogen). At 48 hours post-transfection, H446 and H1975 cells were collected and used for further analyses. The used sequence was listed as below: si-NC sequence (3’-GCGACCAACGCCTTGATTG-5’), si-PCAT6 sequence (3’-GGTGTCTCCATCCTCATTC-5’). the sequences of miR-326 mimic, inhibitor, and respective controls were as followed: miR-326 mimic (5’-ACTGTCCTTCCCTCTGGGC-3’), miR-NC (5’-ACGGTACCCATTTCAGGTA-3’), miR-326 inhibitor (5’-TGACAGGAAGGGAGACCCG-3’); anti-NC (5’-TTGCTAACCTGGTATGCGG-3’).

### 2.10 Bioinformatics analysis and dual-luciferase reporter assay

The online bioinformatics tool starBase v2.0 (http://starbase.sysu.edu.cn/starbase2/) was adapted to predict the potential target genes and conclude the specific binding sites. The relationship between miR-326 and PCAT6 or Wnt5a was verified by dual-reporter assay. In brief, the sequences of PCAT6 wild type (WT) containing the binding sites with miR-326 and corresponding PCAT6 mutant (MUT) sequences were amplified and cloned into the pRL-CMV vector (Promega, Madison, WI, USA), named as PCAT6-WT and PCAT6-MUT. Then, PCAT6-WT and PCAT6-MUT were introduced into H446 and H1975 cells together with miR-326 or miR-NC, respectively. After 48 h, the luciferase activity was detected using the Dual-Luciferase Reporter Assay Kit (Promega). In a similar way, Wnt5a-WT and Wnt5a-MUT were also constructed and used for luciferase activity analysis.

### 2.11 Statistical analysis

Data were collected from at least 3 independent experiments, processed by GraphPad Prism 7.0 (GraphPad software Inc., San Die, CA, USA), and exhibited as mean ± standard deviation (SD). Then Student’s *t*-test was performed to compare the difference between two sets of data which came from two groups, and One-way analysis of variance followed by Tukey’s test was employed to compare the differences among multiple groups. *P* value < 0.05 was regarded as statistically significant.

## 3 Results

### 3.1 Sevoflurane inhibited proliferation, and invasion, but facilitated apoptosis, and deactivation of Wnt/β-catenin signaling pathway in H446 and H1975 cells

**Figure 1:**
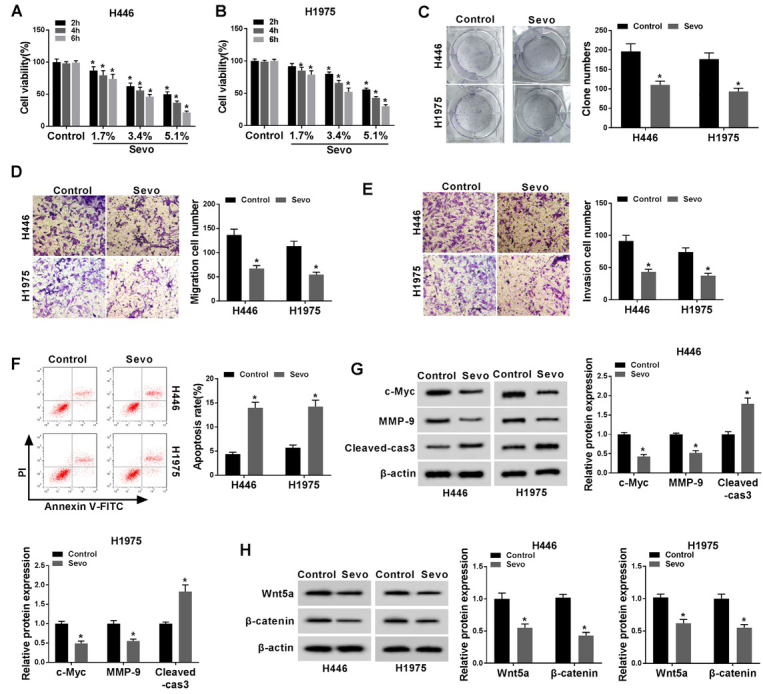
**Sevoflurane inhibited proliferation, and invasion, but facilitated apoptosis, and activation of the Wnt/β-catenin signaling pathway in H446 and H1975 cells**. Cells were exposed to different concentrations (0%, 1.7%, 3.4%, and 5.1%) of sevoflurane for 2h, 4h and 6 h. **(A-B)** The cell viability at determined concentrations and time spans was analyzed by MTT assay in H446 and H1975 cells. The further bio-function assays were performed under the 3.4% concentration of sevoflurane at 6 h. **(C)** Colony formation assay reveals the suppressed proliferating ability through sevoflurane exposure. **(D-E)** The cell migration **(D)** and invasion **(E)** were evaluated by transwell assay. **(F)** The rate of apoptosis was measured by flow cytometry assay. **(G)** Expression levels of c-Myc, MMP-9 and Cleaved-caspase-3 were determined by western blotting assays in H446 and H1975 cells after sevoflurane administration. **(H)** Expression levels of Wnt5a and β-catenin were downregulated by sevoflurane in western blotting assays in H446 and H1975 cells. ^*^P<0.05. Student’s *t*-test was performed to compare the difference between two sets of data which came from two groups, and One-way analysis of variance followed by Tukey’s test was employed to compare the differences among multiple groups. *P* value < 0.05 was regarded as statistically significant.

To observe the bio-function role of sevoflurane in H446 and H1975 cells, the cells were treated with different concentrations with 1.7%, 3.4% and 5.1% of sevoflurane for 2 h, 4 h and 6 h. In several experiments, 5.1% [33, 34] and 4% [35] of sevoflurane was recognized a considered concentration. But, of our experiments, in view of the different types of cell lines we selected, under high concentration conditions, a series of functional experiments are not easily detected owing to cell death caused by sevoflurane, so we chose a lower concentration for subsequent experiments. When treated with 3.4% sevoflurane for 6 hours, the cell proliferation activity was reduced by 50%. Therefore, 3.4% sevoflurane was selected for 6h for subsequent experiments. Compared with the untreated group, the addition of sevoflurane greatly reduced the viability of H446 and H1975 cells in a dose- and time- dependent manner (Figure 1A-B, *P* < 0.05); Taken together, these data indicated that sevoflurane significantly suppressed the viability of lung cancer cells. After that, the 3.4% dosage of sevoflurane was selected for further steps of function assays due to the significant difference with untreated control. Moreover, the colony formation assay demonstrated that sevoflurane exposure limited the cell colony formation in either H446 or H1975 cells, compared with the non-treated group (Figure 1C, *P* < 0.05). Also, we determined the number of migrative and invasive cells using the transwell assay. As shown in Figure 1D-E, *P* < 0.05, sevoflurane was able to significantly decrease the number of H446 and H1975 cells that penetrated the membrane relative to the group control. Furthermore, we analyzed the role of sevoflurane in lung cancer cell apoptosis. The results of the apoptosis assay demonstrated that the apoptotic rate was remarkably increased in H446 and H1975 cells exposed to sevoflurane compared with that in the untreated group (Figure 1F, *P* < 0.05). Meanwhile, sevoflurane significantly inhibited the protein expression of c-Myc and MMP-9, but promoted the expression of Cleaved-caspase-3 in H446 and H1975 cells (Figure 1G, *P* < 0.05). Besides, Wnt/β-catenin signaling-related proteins expression were analyzed in H446 and H1975 cells by Western blot. The results displayed that the levels of Wnt5a and β-catenin expression were markedly decreased in H446 and H1975 cells treated with sevoflurane (Figure 1H, *P* < 0.05).

### 3.2 Sevoflurane inhibited PCAT6 expression and upregulated miR-326 expression in lung cancer cells

**Figure 2:**
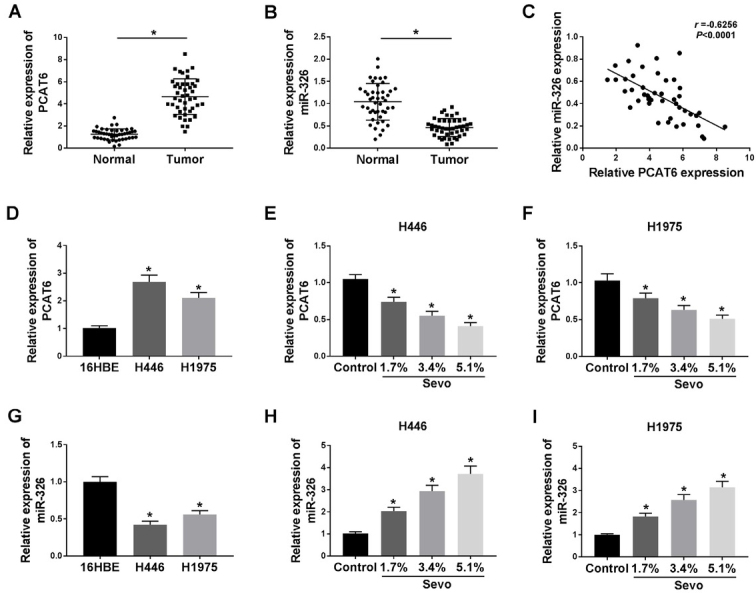
**Sevoflurane inhibited PCAT6 expression and upregulated miR-326 expression in lung cancer cells. (A, B)** qRT-PCR was used to detect the expression of PCAT6 **(A)** or miR-326 **(B)** in tissues from 45 patients with lung cancer. **(C)** A correlation between the expression of miR-326 and PCAT6 was explored. **(D, G)** The expression of PCAT6 **(D)** or miR-326 **(G)** in human bronchial epithelial cells (16HBE) and human lung cancer cell lines (H446, H1975) was measured by qRT-PCR assay. **(E-F)** The expression of PCAT6 was detected in H446 **(E)** and H1975 **(F)** cells being exposed to different concentrations (0%, 1.7%, 3.4%, and 5.1%) of sevoflurane for 6 h. **(H-I)** The expression of miR-326 was measured in H446 **(H)** and H1975 **(I)** cells. ^*^P<0.05. Student’s *t*-test was performed to compare the difference between two sets of data which came from two groups, and One-way analysis of variance followed by Tukey’s test was employed to compare the differences among multiple groups. *P* value < 0.05 was regarded as statistically significant.

To further explore the role of sevoflurane in the molecular regulation of cancer cells, we collected a total of 45 tissue samples, including lung cancer nidus and paired adjacent parts. We filtrated expression of lncRNAs in lung cancer cells after sevoflurane treatment, namely PCAT6, UCA1, SNHG1, CCAT2, and found that the level of PCAT6 was significantly suppressed after sevoflurane administration. As a result, we selected PCAT6 for further study. We measured the expression of PCAT6 and miR-326. Notably, the cancer nidus expressed a higher PCAT6 pattern, or a lower miR-326 tendency, as compared to the corresponding part of tissues (Figure 2A-B, *P* < 0.05). Meanwhile, we analyzed that the expression of PCAT6 negatively correlated with that of miR-326 in tissues (Figure 2C, *P* < 0.05). Not surprisingly, PCAT6 expression was enhanced in the H446 and H1975 cells, with the limitation of miR-326 expression (Figure 2D, *P* < 0.05; 2G, *P* < 0.05). Besides, in the cells, the increased concentration of sevoflurane led to the PCAT6 expressed decrease (Figure 2E-F, *P* < 0.05), and miR-326 promotion (Figure 2H-I, *P* < 0.05), compared with their un-administrated controls.

### 3.3 Overexpression of PCAT6 reversed the effects of sevoflurane on H446 and H1975 cells

**Figure 3:**
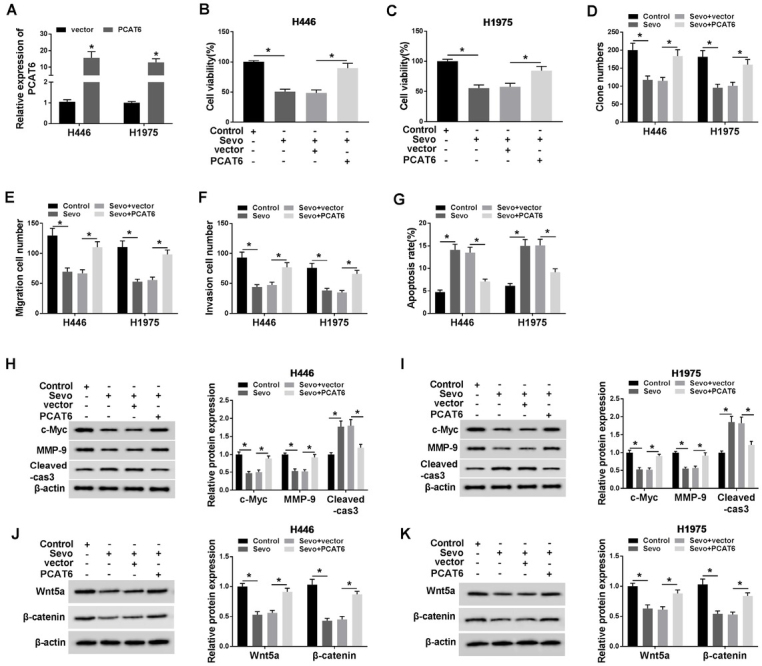
**Overexpression of PCAT6 reversed the effects of sevoflurane on H446 and H1975 cells. (A)** The efficiency of PCAT6 overexpression was shown. **(B-G)** It was shown that restoration by PCAT6 overexpression of cells upon cell viability **(B-C)**, proliferation **(D)**, migration **(E)**, invasion **(F)**, apoptosis **(G)**. **(H-I)** PCAT6 overexpression inverted the protein expression (c-Myc, MMP-9 and Cleaved-caspase-3) induced by sevoflurane in H446 and H1975 cells. **(J-K)** PCAT6 overexpression inverted the protein expression (Wnt5a and β-catenin) induced by sevoflurane in H446 and H1975 cells. ^*^P<0.05. Student’s *t*-test was performed to compare the difference between two sets of data which came from two groups, and One-way analysis of variance followed by Tukey’s test was employed to compare the differences among multiple groups. *P* value < 0.05 was regarded as statistically significant.

Subsequently, we overexpressed the PCAT6 expression in two different cells, and its expression was around 10-fold higher than before, being confirmed by qRT-PCR assay (Figure 3A, *P* < 0.05). A series of physiological exploration was conducted by MTT assay, colony formation assay, migration/invasion transwell assay, and flow cytometry, individually. The MTT and colony formation results showed that the upregulation of PCAT6 limited the suppressing effects of sevoflurane on H446 and H1975 cell proliferation (Figure 3B-D, *P* < 0.05). As shown in Figure 3E-F, *P* < 0.05, the upregulation of PCAT6 abolished the inhibitory effects of sevoflurane on cell migration and invasion. In addition, the overexpression of PCAT6 reversed the effects of sevoflurane on increased cell apoptosis in H446 and H1975 cells (Figure 3G, *P* < 0.05). Besides, PCAT6 reversed the blocked protein expression of c-Myc and MMP-9 and increased protein expression of Cleaved-caspase-3 in sevoflurane-treated cells (Figure 3H-I, *P* < 0.05). Moreover, the Western blot analysis revealed that H446 and H1975 cells transfected with PCAT6 also prevented the decrease of Wnt5a and β-catenin protein expression caused by sevoflurane treatment (Figure 3J-K, *P* < 0.05).

### 3.4 MiR-326 was the target of PCAT6

**Figure 4:**
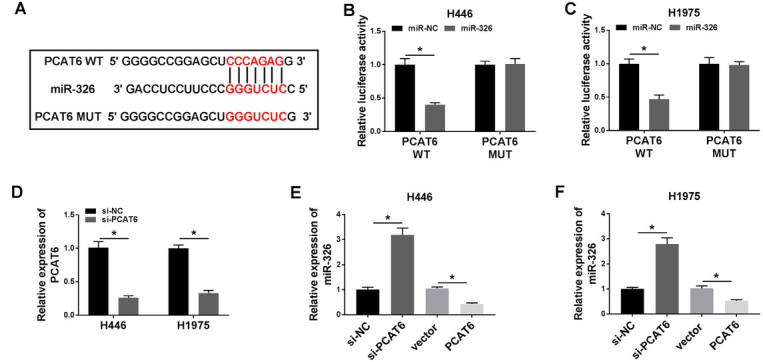
**MiR-326 was the target of PCAT6. (A)** The putative binding sites between miR-326 and PCAT6 were predicted by starBase v2.0. **(B-C)** The predicted sites were identified by dual-luciferase reporter assay. **(D)** The expression of PCAT6 in H446 or H1975 cells were detected by qRT-PCR after PCAT6 knockdown. **(E-F)** The expression of miR-326 in H446 **(E)** or H1975 **(F)** cells was detected by qRT-PCR after PCAT6 knockdown or overexpression. ^*^P<0.05. Student’s *t*-test was performed to compare the difference between two sets of data which came from two groups. *P* value < 0.05 was regarded as statistically significant.

Previous investigations have demonstrated that lncRNAs serve as competing endogenous RNAs (ceRNAs) and sponge miRNAs, regulating the expression of target mRNA. To find out the specific miRNA that was regulated by PCAT6, we performed bioinformatics analysis and dual-luciferase reporter assay. First, the results of the bioinformatics analysis starBase (http://starbase.sysu.edu.cn/index.php) suggested that miR-326, which contained the putative binding sites, was the target of PCAT6 (Figure 4A). In order to further prove miR-326 was the target of PCAT6, the dual-luciferase reporter assay was applied. The fragment of PCAT6 containing miR-326 binding sites (WT-PCAT6) or mutant fragment (MUT-PCAT6) was inserted into plasmid. Then, cells were co-transfected with plasmid and miR-326 or miR-NC. As shown in Figure 4B-C, *P* < 0.05, the luciferase activity in WT-PCAT6-transfected cells was significantly decreased by miR-326. However, there was no change in cells that was transfected with MUT-PCAT6. Next, the level of miR-326 was decreased in H446 and H1975 cells transfected with si-PCAT6 for silencing experiments (Figure 4D, *P* < 0.05). After that, we assessed the level of miR-326 in lung cancer cells that were transfected with si-PCAT6 or PCAT6 vector, and we found that the level of miR-326 was markedly increased in PCAT6 down-expressing H446 and H1975 cells or decreased in PCAT6 up-expressing H446 and H1975 cells (Figure 4E-F, *P* < 0.05).

### 3.5 Overexpression of miR-326 reversed the effects of PCAT6 upregulation in sevoflurane-induced lung cancer cells in vitro

**Figure 5:**
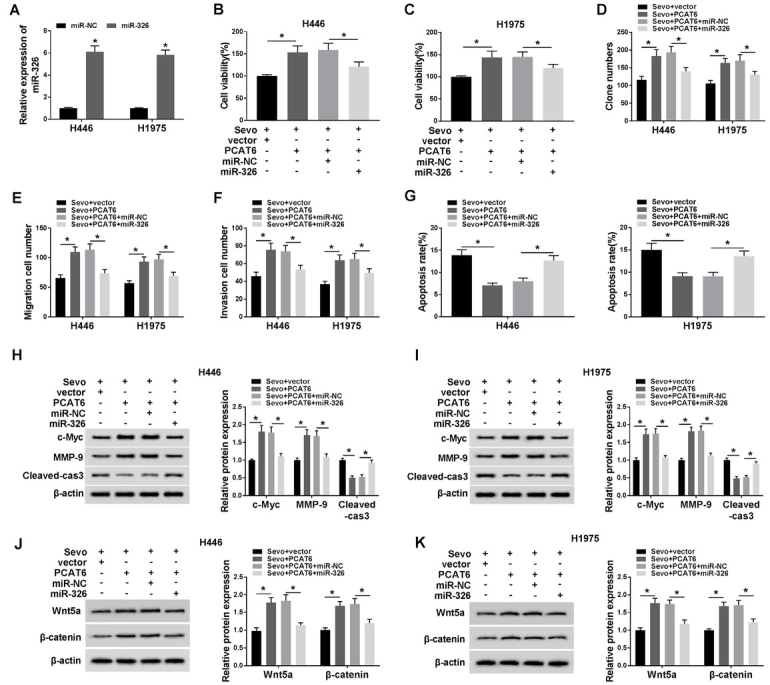
**Overexpression of miR-326 reversed the effects of PCAT6 upregulation in sevoflurane-induced lung cancer cells *in vitro*. (A)** The expression of miR-326 was restored in sevoflurane-administrated cells by transfecting with PCAT6 and miR-326. A serial of physiological assays was conducted to assess the cell viability **(B-C)**, proliferation **(D)**, migration **(E)** invasion **(F)**, and apoptosis **(G)** in cells by miR-326 recuperation. **(H-I)** The protein patterns (c-Myc, MMP-9 and Cleaved-caspase-3) were inverted by miR-326 recuperation in H446 **(H)** and H1975 **(I)** cells. **(J-K)** Wnt5a and β-catenin protein patterns were inverted by miR-326 recuperation in H446 **(J)** and H1975 **(K)** cells. ^*^P<0.05. Student’s *t*-test was performed to compare the difference between two sets of data which came from two groups, and One-way analysis of variance followed by Tukey’s test was employed to compare the differences among multiple groups. *P* value < 0.05 was regarded as statistically significant.

To further confirm whether the promoted effects of PCAT6 on biological functions were mediated by miR-326 in sevoflurane-induced cells, we transfected PCAT6, PCAT6+miR-NC, or PCAT6+miR-326 into sevoflurane-induced cells. First, the expression of miR-326 of cells was successfully elevated by miR-326 transfection (Figure 5A, *P* < 0.05). Secondly, the upregulation of miR-326 abolished the facilitated effects of PCAT6 promotion on H446 and H1975 cells viability (Figure 5B-C, *P* < 0.05) and proliferation (Figure 5D, *P* < 0.05). Thirdly, the up-expression of miR-326 reversed the effects of PCAT6 on induced cell migrative and invasive ability in H446 and H1975 cells (Figure 5E-F, *P* < 0.05). Also, the overexpression of miR-326 reversed the effects of PCAT6 on decreased cell apoptosis in H446 and H1975 cells (Figure 5G, *P* < 0.05). Meanwhile, detection of western blot showed that miR-326 rescued the upward tendency (c-Myc and MMP-9) and downward tendency (Cleaved-caspase-3) in PCAT6 and sevoflurane induced cells (Figure 5H-I, *P* < 0.05). Moreover, the western blot analysis revealed that H446 and H1975 cells transfected with miR-326 also inverted the increase of Wnt5a and β-catenin protein expression caused by PCAT6 enforced treatment (Figure 5J-K, *P* < 0.05). These findings indicated that the overexpression miR-326 could restore the effects of PCAT6 on cell proliferation, invasion, apoptosis, and the activation of Wnt/β-catenin signaling in H446 and H1975 cells.

### 3.6 Wnt5a was a direct target of miR-326

**Figure 6:**
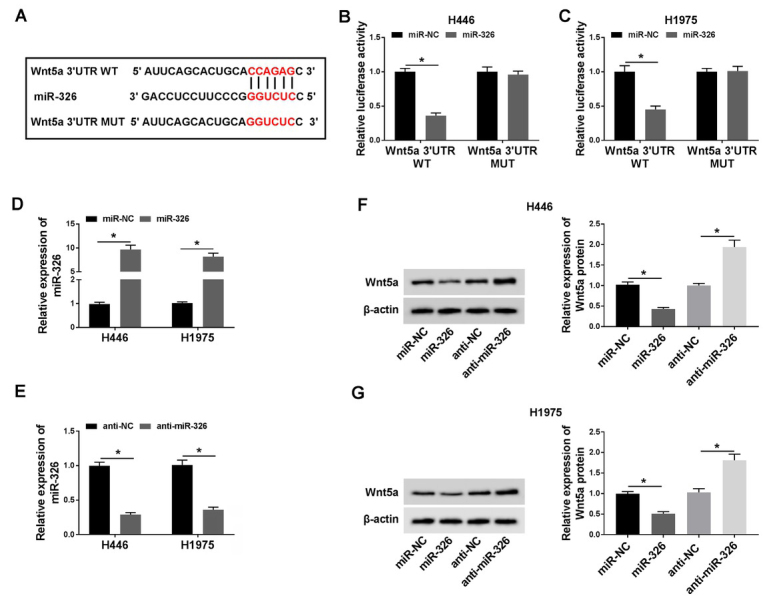
Wnt5a was a direct target of miR-326. **(A)** The putative binding sites between miR-326 and PCAT6 were predicted by starBase v2.0. **(B-C)** The predicted sites were identified by dual-luciferase reporter assay. **(D-E)** The expression of miR-326 in cells was detected by qRT-PCR after miR-326 overexpression **(D)** or knockdown **(E)**. **(F-G)** The expression of Wnt5a was explored by western blot after H446 **(F)** and H1975 **(G)** cells transfected with miR-326 or anti-miR-326. ^*^P<0.05. Student’s *t*-test was performed to compare the difference between two sets of data which came from two groups. *P* value < 0.05 was regarded as statistically significant.

StarBase v2.0 was utilized to identify the potential targets of miR-326 on the 3’-UTR of Wnt5a mRNA containing the putative binding site for miR-326 (Figure 6A). Following the dual-luciferase activity, the assay was carried out to further confirm the target relationship between miR-326 and Wnt5a. Results demonstrated that the luciferase activity of Wnt5a-WT was inhibited by miR-326, as no distinct change of Wnt5a-MUT luciferase activity was detected (Figure 6B-C, *P* < 0.05). Afterward, we constructed the overexpressed miR-326 and anti-miR-326 vectors and the efficiency was assessed by qRT-PCR. It could be seen that the expression of miR-326 was probably 10 times larger when cells transfected with miR-326, with half of miR-326 level after anti-miR-326 transfection, compared with their negative controls (Figure 6D-E, *P* < 0.05). The protein level of Wnt5a in H446 and H1975 cells was measured by western blot assay and the results showed that the Wnt5a level was down-regulated by miR-326, or up-regulated by anti-miR-326 (Figure 6F-G, *P* < 0.05).

### 3.7 Overexpression of Wnt5a reversed the effects of miR-326 on lung cancer cells

**Figure 7:**
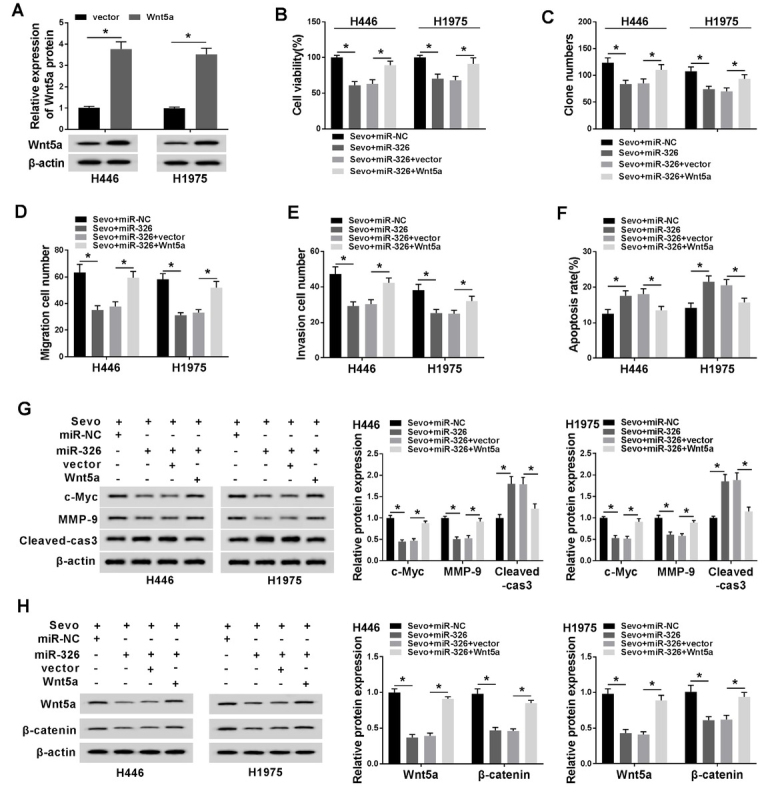
**Overexpression of Wnt5a reversed the effects of miR-326 in lung cancer cells. (A)** The efficacy of Wnt5a overexpression was verification by western blot. The sevoflurane-induced H446 and H1975 cells were co-transfected with miR-326 and Wnt5a. **(B-F)** The cell viability **(B)**, proliferation **(C)**, migration **(D)** invasion **(E)**, and apoptosis **(F)** were investigated by MTT analysis, transwell invasion assay, flow cytometry assay individually. **(G)** Overexpression of Wnt5a in cells partly reversed miR-326-induced levels of c-Myc, MMP-9 and Cleaved-caspase-3, which was assessed by western blot. **(H)** Overexpression of Wnt5a in cells partly reversed miR-326-induced levels of Wnt5a and β-catenin, which was assessed by western blot. ^*^P<0.05. Student’s *t*-test was performed to compare the difference between two sets of data which came from two groups, and One-way analysis of variance followed by Tukey’s test was employed to compare the differences among multiple groups. *P* value < 0.05 was regarded as statistically significant.

To figure out whether Wnt5a worked downstream of miR-326, the experimental groups were designed, namely sevo+miR-NC, sevo+miR-326, sevo+miR-326+vector, and sevo+miR-326+Wnt5a. Through Wnt5a overexpression transfection, Wnt5a transfection triggered a promotion on protein expression of Wnt5a in sevoflurane-administrated H446 and H1975 cells (Figure 7A, *P* < 0.05). Wnt5a transfection inverted the limited viability (Figure 7B, *P* < 0.05) and proliferation (Figure 7C, *P* < 0.05) of cells transfected with miR-326 as well. After that, Wnt5a overexpression re-facilitated the low-migratory/invasion ability (Figure 7D-E, *P* < 0.05), but re-blocked the pro-apoptosis ability (Figure 7F, *P* < 0.05) due to miR-326 transfection. Also, Upregulation of miR-326 in H446 and H1975 cells could restrain the protein expression (c-Myc and MMP-9) and enhance the Cleaved-caspase-3 expression, it could be restored by Wnt5a transfection (Figure 7G, *P* < 0.05). Upregulation of miR-326 in H446 and H1975 cells could suppress the protein expression of Wnt5a and β-catenin; and it could be restored by Wnt5a transfection as well (Figure 7H, *P* < 0.05).

## 4 Discussion

Lung cancer is the most common malignant cancer worldwide, and a large number of which are non-small cell lung cancer, accounting for 80 percent [36]. Metastasis with high occurrence after lung cancer surgery ranks as the leading cause of death in lung cancer patients [37, 38]. Thus, it was essential to identify a more effective and novel molecular prognostic indicator for lung cancer. Sevoflurane, a volatile anesthetic agent, was used widely during lung cancer surgery. The anti-migration and invasion effect of sevoflurane on lung cancer A549 cells and papillary thyroid carcinoma (TPC-1 and IHH-4) cells has been demonstrated in two studies [1, 2].

Upregulation of PCAT6 was found in colorectal cancer [39], cervical cancer [40], and lung cancer [16]. Our results revealed that PCAT6 was upregulated in lung cancer tissues and cells, which was induced by sevoflurane, and overexpression of PCAT6 inverted the sevoflurane’s repression on viability, proliferation, invasion, and promotion on apoptosis of human lung cancer cells *in vitro*. Besides, the overexpression of PCAT6 inverted the inactivation of the Wnt/β-catenin pathway of sevoflurane. Analogously, Lǚ *et al* and his colleagues found that PCAT6 enhanced cell growth and metastasis by Wnt/β-catenin pathway in cervical cancer [40].

Accumulative evidence documented that lncRNAs regulated the expression of target mRNAs by acting as competitive endogenous RNAs (ceRNAs) to sponge miRNAs. Based on the above studies, the sponging relationship between PCAT6 and miR-326 was verified through the dual-luciferase reporter assay. Moreover, PCAT6 upregulation mediated-effects on viability, proliferation, invasion, apoptosis, and promotion of Wnt/ β-catenin pathway were reversed by introducing with miR-326.

Next, we also investigated plenty of findings to clarify the function of miR-326 in the occurrence and development of human cancers. Some studies were conducted and indicated that miR-326 might serve as a tumor inhibitor in lung cancers [21, 22]. We observed miR-326 expression was decreased in tumor tissues, could be upregulated by sevoflurane, and PCAT6 partly modulated lung cancer progression by sponging miR-326. Thus, we investigated the role of miR-326 on lung cancer cells *in vitro*, research results indicated that Wnt5a was directly targeted by miR-326 and a significant inverse correlation between them was also confirmed. What’s more, a functional experiment suggested that the upregulation of miR-326 counteracted the impacts of PCAT6 expression on viability, proliferation, invasion, apoptosis, and activation of the Wnt/β-catenin pathway in lung cancer cells.

**Figure 8:**
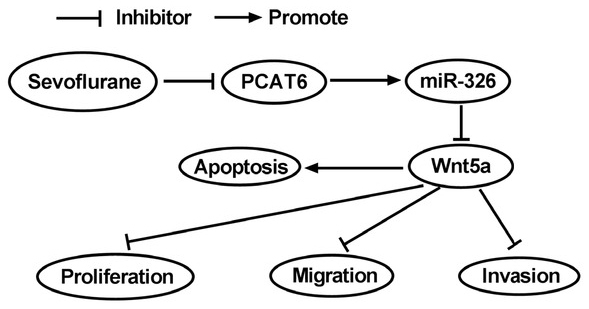
**regulatory diagram of mechanism of sevoflurane**. Regulatory networks among PCAT6, miR-326, and Wnt5a after sevoflurane administration.

As for Wnt5a, an activator of Wnt/β-catenin pathway, played a critical role in the progress and development in many malignant tumors [41]. For instance, it was described that the enforced expression of Wnt5a promoted epithelial-to-mesenchymal transition and metastasis in non-small-cell lung cancer [42]. The functional experiment of this part revealed that Wnt5a declined in lung cancer cells introduced with miR-326, and its expression was increased with miR-326 inhibitor, suggesting Wnt5a was negatively regulated by miR-326 in lung cancer. Moreover, overexpression of Wnt5a overturned the effects on viability, proliferation, invasion, apoptosis, and Wnt/β-catenin restraint of miR-326 expression in lung cancer cells. The limitation of this experiment was that the commercial cell lines we selected did not fully explain the tumor-suppressive effects of sevoflurane on all kinds of cells. In addition to the dual-luciferase reporter assay, the targeting relationship required further proof including RNA Immunoprecipitation and RNA pull-down experiments. In conclusion, we discovered that sevoflurane could inhibit lung cancer progression *in vitro*. Mechanism analysis revealed that sevoflurane suppressed the Wnt5a expression in lung cancer cells by regulating PCAT6 sponging miR-326, suggesting that sevoflurane modulated PCAT6/miR-326/Wnt5a/β-catenin loops to restrain lung cancer process and compensated for the research gap in the anti-tumor mechanism of sevoflurane.
